# Using costing to facilitate policy making towards Universal Health Coverage: findings and recommendations from country-level experiences

**DOI:** 10.1136/bmjgh-2022-010735

**Published:** 2023-01-18

**Authors:** Sylvestre Gaudin, Wajeeha Raza, Jolene Skordis, Agnès Soucat, Karin Stenberg, Ala Alwan

**Affiliations:** 1 Department of Economics, Oberlin College, Oberlin, Ohio, USA; 2 Centre for Health Economics, University of York, York, UK; 3 Centre for Global Health Economics, University College London, London, UK; 4 Division of Health and Social Protection, French Development Agency (AFD), Paris, France; 5 Department of Health Systems Governance and Financing, World Health Organization, Geneva, Switzerland; 6 Swiss Tropical and Public Health Institute, Allschwil, Switzerland; 7 University of Basel, Basel, Switzerland; 8 DCP3 Country Translation Project, London School of Hygiene & Tropical Medicine, London, UK

**Keywords:** health policy, health economics, cross-sectional survey

## Abstract

As countries progress towards universal health coverage (UHC), they frequently develop explicit packages of health services compatible with UHC goals. As part of the Disease Control Initiative 3 Country Translation project, a systematic survey instrument was developed and used to review the experience of five low-income and lower-middle-income countries—Afghanistan, Ethiopia, Pakistan, Somalia and Sudan—in estimating the cost of their proposed packages. The paper highlights the main results of the survey, providing information about how costing exercises were conducted and used and what country teams perceived to be the main challenges. Key messages are identified to facilitate similar exercises and improve their usefulness. Critical challenges to be addressed include inconsistent application of costing methods, measurement errors and data reliability issues, the lack of adequate capacity building, and the lack of integration between costing and budgeting. The paper formulates four recommendations to address these challenges: (1) developing more systematic guidance and standard ways to implement costing methodologies, particularly regarding the treatment of health systems-related common costs, (2) acknowledging ranges of uncertainty of costing results and integrating sensitivity analysis, (3) building long-term capacity at the local level and institutionalising the costing process in order to improve both reliability and policy relevance, and (4) closely linking costing exercises to public budgeting.

**Summary box**

- There is a wide variation in the way costing methodologies are implemented and terminology interpreted, particularly regarding common health system-related costs and capacity constraints, calling for more systematic guidance.
- Estimating the costs of essential packages of health services (EPHS) is not an exact science. In order to ensure policy relevance, improve confidence in results and foster effective use in decision making, costing needs to integrate sensitivity analysis, acknowledge realistic ranges of uncertainty and be regularly updated.
- Costing EPHS is a challenging exercise that requires specific skills and expertise. Short-term one-off training is helpful but not sufficient—building long-term institutional capacity is needed for better reliability and policy relevance.
- Costing and budgeting of EPHS are currently separate processes, thereby hindering implementation. Costing exercises need to be designed to support budgetary processes and facilitate budget decisions.

## Introduction

In 2015, all UN Member States adopted the Sustainable Developments Goals (SDG), including SDG target 3.8 on achieving universal health coverage (UHC).^1^ Since then, in their effort to achieve UHC, low-income and lower middle-income countries (LLMICs) have been engaged in processes to define essential packages of health services (EPHS). Estimating implementation costs is a critical step in package design.^2 3^ Countries need to produce reliable cost estimates to facilitate effective communication with policy makers, health budget dialogue and mobilisation of proper financing. High-quality cost estimates can help ensure that an EPHS is not simply aspirational but feasible for implementation within a country’s budget.

In this paper, we review the experiences of five LLMICs—Afghanistan, Ethiopia, Pakistan, Somalia and Sudan—in estimating EPHS costs and using cost results in decision making. Our goal is not to evaluate or compare the output of costing but to focus on processes and identify challenges that countries faced, so as to facilitate future exercises and improve their usefulness.

The paper is part of a series on the experience of six LLMICs in designing their own EPHS. Alwan *et al*^4^ provide a broad outline of the aims of the series as well as summary information on package characteristics and package development process in the six countries. All countries are part of an extended knowledge network regrouping LLMICs that had developed or are developing an EPHS, at least partially inspired by evidence from the third edition of the Disease Control Priorities (DCP3).^4 5^ For more complete information on the countries’ costing exercises, package content and quantitative costing results, we refer the reader to country-specific publications and/or reports.^6–9^

Evidence for this paper was gathered using a systematic survey aimed at technical costing teams. The survey questionnaire is presented in online supplemental annex 1. It includes 28 factual and 10 normative questions on the choice of methodology, capacity, data issues and the use of costing estimates for policy and planning decisions. Instructions were provided with guidance on who should be involved in responses (one person identified as primary contact, but participation of the entire costing team encouraged), how responses would be used (countries named only on factual questions and respondents never identified by name) and terminology. The instrument was reviewed by health economists and public health experts with prior experience in costing for package development. Our questions on methodologies and tools were designed to match elements included in a 2021 systematic literature review of costing EPHS in low-income and middle-income countries.^10^ The questionnaire was sent in December 2021 to all LLMICs within the DCP3 network that had recently carried out and completed the costing of their EPHS—a total of six countries. Responses were received from five countries. Respondents indicated that completing the survey required 1–2 hours. Follow-up interviews (10–20 min calls and/or clarification requests via email) ensured that the questions were uniformly understood and that responses were interpreted as the country team intended. The data collection process was completed mid-January 2022.

10.1136/bmjgh-2022-010735.supp1 Supplementary data

There are several other studies describing the methods and results of costing exercises in the context of developing UHC packages^11–13^ or more generally to inform cost-effectiveness analysis,^14 15^ including multicountry studies.^10 16^ However, these studies are either based on systematic reviews of the literature^15 16^ or report on the authors’ knowledge and experience.^11–13^ Our study is the first to capture and compare experiences from the perspective of local costing teams, fully based on their feedback, and to consolidate these reflections into key recommendations for future costing exercises.

## Summary of country experiences in costing their EPHS

Full survey results are presented in online supplemental annex 2. Reported results are those provided by survey respondents and, as such, are indicative of the teams’ knowledge of the exercise rather than any country’s official position.

10.1136/bmjgh-2022-010735.supp2 Supplementary data

### Scope and timing

All countries included both individual and population-based health interventions in the same costing exercise; none of the five countries estimated costs of intersectoral interventions (online supplemental annex 2, table Q1). Some packages were limited to primary healthcare, while others included hospital care. Costing exercises were initiated in 2018 in Ethiopia and Pakistan, in 2019 in Afghanistan and Somalia, and in 2020 in Sudan; they lasted a minimum of 6 months in Afghanistan and up to 20 months in Somalia (online supplemental annex 2, table Q3).

### Costing teams: composition, skills and capacity building

Team sizes varied widely (table 1). Most countries relied heavily on Ministry of Health (MoH) staff with some support from international consultants, although we note important differences. Pakistan is the only country that directly involved a local university in addition to MoH staff and external consultants. All countries involved international experts, although their involvement was only peripheral in two countries.

**Table 1:** Composition of the costing teams

From Q3 and Q5 (online supplemental annex 1)	Mean	Afghanistan	Ethiopia	Pakistan	Somalia	Sudan
Core team—no of people	7	2	8	5	8	12
Of which: Ministry of Health/government	62%	100%	50%	20%	50%	92%
International experts/external partners/academia	22%	0%	38%	20%	50%	0%
Local schools/universities	12%	0%	0%	60%	0%	0%
Other within country	4%	0%	13%	0%	0%	8%
Peripheral*— no of people	13	3	30	5	16	11
Of which: Ministry of Health/government	47%	67%	50%	40%	25%	55%
International experts/external partners/academia	33%	33%	17%	20%	50%	45%
Local schools/universities	13%	0%	17%	40%	6%	0%
Other within country	7%	0%	17%	0%	19%	0%

*Includes people who were not part of the costing team but worked on specific requests from the team, such as providing intervention descriptions, estimates of population in need, current budget numbers, etc. Also includes supervisors, expert reviewers, support staff, etc.

The proportion of core team members with no pre-existing costing skills was high, ranging from 50% in Afghanistan and Somalia to 83% in Sudan. In Somalia, there was no specific training other than on-the-job training—that is, learning by doing with guidance from skilled members. All countries but Somalia received technical support from development partners and three received specific training for the costing exercise, mostly locally (online supplemental annex 2, table Q5).

The perceived value of different types of capacity building was assessed for the current and future costing exercises (online supplemental annex 2, table Q34). On-the-job training was ranked highest in all countries but all training categories received average ratings above 7.5 and at least one highest rating of 10, indicating they were all considered useful. ‘Classes and workshops organised abroad’ was the only category that received an average evaluation below 8 and one rating below 5, although two countries gave it the highest rating. Two types of skills were specifically requested: capacity in health economics and practical analytical skills to handle costing tools.

### Costing methods and tools used

All five countries surveyed used bottom-up ingredient-based methods to estimate the costs of healthcare interventions on the person (online supplemental annex 2, table Q8). Expenditure-based top-down approaches were only used for population-based interventions in Pakistan and for specific disease programmes in Sudan.

A variety of tools could be used to estimate costs and to analyse and present results. Many different possible tools were included in Q9 of the survey (online supplemental annex 1) but only a few were applied (online supplemental annex 2, table Q9). Excel-based spreadsheets were the most commonly used—it was the main estimation tool in Pakistan—while all countries but one (Somalia) employed them for analysis and presentation. The OneHealth Tool (OHT)^17^ was used in Ethiopia, Somalia and Sudan, and the Cost Revenue Analysis (CORE) Plus tool^18^ was used in Afghanistan. The HIPtool^19^ was used to complement spreadsheets for analysis and presentation in Pakistan.

We asked countries what features they most appreciated in the tools they used (online supplemental annex 1, Q31; responses in online supplemental annex 2, table Q31). The OHT was viewed as effective in procuring high-quality estimates due to its comprehensive treatment of different aspects of health system-related costs; but the process was reported as time-consuming and data-demanding. Two countries highlighted the OHT functionalities to present and analyse results, while another country appreciated its bottleneck and impact analysis. One country viewed the CORE Plus tool as highly effective in facilitating costing using bottom-up methods and in helping to procure both normative and real-world estimates. The bookshelf and other visualisation tools provided by the HIPtool received special mention by one country as facilitating the analysis and presentation of results.

Methodological choices were mostly influenced by the countries’ health sector planning methods and governance structures but only two countries took into account public budgeting methods and national plans (online supplemental annex 2, table Q7). It is clear that these choices were influenced by ex-ante goals, examined later. Practical considerations in choosing methodology and tools highlighted the ‘skills/capacity required’ followed by ‘availability/accessibility of specific guidance’ and ‘time/data considerations’ (online supplemental annex 2, table Q10). The guidance provided by these tools was deemed satisfactory overall, except for the evaluation of time and data requirements (online supplemental annex 2, table Q29). The need for flexibility and transparency also motivated the decision not to use tools such as OHT in one country, where Excel spreadsheets were used instead.

### Data sources used in costing

Different types of data were used in costing depending on costing methodology: quantity and price data for bottom-up methods; expenditure data for top-down approaches; and health and population data to evaluate target populations. Four out of five countries reported on the data sources. In all countries, national government sources were most used—predominantly data routinely collected by ministries of health—but they had to be largely complemented by additional sources, including primary data collection in two countries (online supplemental annex 2, tables Q18 and Q19).

The variety of complementary sources listed in most countries for quantity and price data, and the fact that some countries had to resort to time-consuming primary data collection and/or searches through local cost studies and reports show that data collection was a challenge, particularly when using bottom-up methods.

Estimations of target populations were primarily sourced from health management information systems, global burden of disease data, demographic and health surveys, and other national surveys (online supplemental annex 2, table Q20). In case of data gaps, literature reviews and expert advice were used. One country simply assumed current coverage (utilisation) at 10% of the population when other data were not available.

### Implementation of costing methodology

As primary inputs to calculate direct costs, all countries surveyed included human resources, drugs/medicines, other materials and supplies, and medical equipment (online supplemental annex 2, table Q12). In some cases, unit costs of subservices were used instead of primary inputs for inpatient costs, radiology/diagnostic services and laboratory services. Overhead was included by four countries, and building use, vehicles, management and supply chain costs were considered by three countries.

When asked to describe how indirect (common) health system costs were incorporated, three countries indicated that they were automatically allocated at the intervention level by the tools they used (OHT or CORE Plus); one country used a uniform multiplier of 1.6 (all intervention costs were inflated by 60%), and another indicated that health system-related costs were not systematically included. In relation to capital costs, three out of five countries reported including ‘investment costs to increase general capacity of the health system (human capital, facilities, etc)*‘*, but only one country indicated having used a long-run perspective, which was defined as ‘considering the possibility of significant upgrading and investment in new facilities’.

### Estimated package costs

Information on costing results was collected for the purpose of gauging the extent to which estimates varied within and across countries. Countries reported on total estimated package costs for their aspirational and/or immediate implementation packages (see Alwan *et al*^4^ for information on the different packages), and provided some information on coverage and scope (online supplemental annex 2, table Q2). All countries but Ethiopia indicated that they reported on incremental costs (online supplemental annex 2, table Q23), but no estimates were collected in this survey. Three countries provided figures for progressive implementation plans up to 2030, with coverage of interventions varying from levels below 80% to full population.

Reported per capita package costs show wide ranges. As expected, lowest per capita costs are reported for immediate implementation primary care packages. We caution the reader about using these results for evaluation purposes or to compare across countries. Not only did countries declare different purposes and use different methodologies but the interventions included and the delivery platforms targeted varied considerably.^4–9^

### Sensitivity analysis

Table 2 lists the types of sensitivity analysis that were considered in at least one country. Two countries did not perform any sensitivity analysis and one country only considered different scenarios on extent of coverage (Ethiopia). Somalia and Pakistan presented different scenarios on human resources for health and service coverage. Sensitivity to different data specification and assumptions were only carried out in Pakistan, while Somalia carried out scenarios using different investment plans and geographical expansions.

**Table 2:** Sensitivity/uncertainty analysis and scenarios for EPHS costing

From Q25 (online supplemental annex 1)	No of countries
Any type of sensitivity analysis/scenarios conducted (including listed below)	3
Different scenarios on human resources for health	2
Different scenarios on extent of coverage	2
Different scenarios on investment plans	1
Different geographical expansions	1
Sensitivity to different data specification (eg, using different sources for prices or recommended protocols, using average versus marginal prices)	1
Sensitivity to different assumptions (eg, discount rates, amortisation, available technology, linear/non-linear relationships)	1

EPHS, essential package of health services.

### Utilisation of results in decision making

Countries were asked to describe the stated goals of the exercise (online supplemental annex 1, Q6). General purposes included in their responses are listed in table 3 from most to least frequent. We note that none of the countries specifically mentioned ‘budgeting’ as a purpose (full responses in online supplemental annex 2, table Q6).

**Table 3:** Stated goals of EPHS costing (ex ante)

Categories of goals*	No of countries	Afghanistan	Ethiopia	Pakistan	Somalia	Sudan
Resource needs/affordability	3		x	x		x
Resource mobilisation	3	x	x		x	
Reprioritisation based on resource constraint	3		x	x		x
Demonstrating value for money—efficiency	2	x			x	
Planning resource allocation	1	x				
Contracting	1	x				

*These are extracted from responses to Q6 (online supplemental annex 1). Full text responses are reported in online supplemental annex 2, table Q6.

EPHS, essential package of health services.

Beyond stated goals, we asked about actual and potential uses of the costing exercises in policy making and decision making (online supplemental annex 1, Q27). Costing results had most impact on providing evidence for advisory committee meetings, prioritising interventions, creating implementation scenarios and developing investment cases for funding, the latter indicating that the exercise was geared towards external funders (table 4).

**Table 4:** Principal uses of EPHS costing outputs for different types of policy making/decisions

From responses to Q27 (online supplemental annex 1)	Mean score*	Afghanistan	Ethiopia	Pakistan	Somalia	Sudan
Advisory committee meetings	2.2	3	3	2	3	0
Prioritisation of interventions	1.8	1	3	2	3	0
Implementation scenarios (packages)	1.8	1	3	1	3	1
Business case/resource gaps for funders	1.8	2	3	0	3	1
Service delivery planning	1	2	0	1	2	0
Health workforce planning	1	1	0	0	3	1
Contracting-out (PPPs, NGOs, etc)	1	3	0	0	2	0
Annual budgeting—national/local	0.8	3	0	0	1	0
Medicines, equipment, supplies procurement	0.8	1	0	0	3	0
Implementation at different levels	0.6	1	0	0	2	0
Infrastructure investment plans	0.6	0	0	0	3	0
Quality evaluation of current services	0.6	1	0	0	2	0
Long-term budgeting—national/local	0.4	1	0	0	1	0

*The score is obtained by attributing a value of 3 when the exercise had been determinant, 2 when it had been useful, 1 when it was planned to be used, and 0 when it had neither been used not was planned to be used.

EPHS, essential package of health services; NGOs, non-governmental organisations; PPPs, Public-private partnerships.

Afghanistan is the only country where costing results were reported as determinant for annual budgeting. Three out of five countries did not use or planned to use the EPHS costing for either annual or long-term budgeting. In fact, when asked to evaluate actual and potential uses (table 5), respondents rated the usefulness of EPHS costing in government budgeting as relatively low, a striking finding. Respondents were more enthusiastic about the use of costing results for contracting-out and procurement.

**Table 5:** Assessment of actual and potential uses of EPHS costing in decision making

From responses to Q33 (online supplemental annex 1) Rating scale 0–10	This time		Future exercises	
	N*	Mean rating	N*	Mean rating
Contracting-out (PPPs, NGOs, etc)	4	7.25	4	8.75
Medicines, equipment, supplies procurement	4	7.5	4	8
Prioritisation of health interventions	5	6.8	5	7.4
Service delivery planning	4	6.25	4	7.25
Long-term budgeting—national/local	4	5.5	4	6.75
Infrastructure investment plans	4	4.5	4	5

*N indicates the number of countries that expressed an opinion of a given type of use (instructions indicated to leave blank if a respondent had no opinion or did not know).

EPHS, essential package of health services; NGOs, Non-governmental organisations; PPPs, Public-private partnerships.

### Main perceived challenges and recommendations for future exercises

Top challenges encountered during the costing process were reported as being the time required to carry out the exercise, as well as data scarcity and quality issues (online supplemental annex 2, table Q35). Next were the lack of a reliable assessment of health system capacity and investment needs (rated 8 and above in 4 countries) followed by inadequate skills and expertise in costing. Other challenges that received at least one top rating (10) were the treatment of investment costs, the availability and timeliness of capacity building, availability/accessibility of specific guidance for costing tools, understanding of terminology and COVID-19-related issues. Difficulties in understanding terminology received the lowest average rating, although important differences in interpretation were implicit in the analysis of survey responses.

Finally, countries were asked to give recommendations for future exercises. Responses are reported thematically in online supplemental annex 2, Table Q36-38). Emphasis was placed on the need for training, including for people outside of costing teams. Suggestions on methods pointed to the need to go beyond intervention costing to consider programme levels and heathcare settings as well as capacity constraints. Respondents also pointed out the need to better plan the exercise ex ante and to validate results.

## Key messages and recommendations to improve and facilitate future costing exercises

Estimating the costs of health interventions requires substantial time and effort. It is a waste of resources to conduct such an exercise unless policy makers can use the results. The survey responses revealed important differences in the framing of the exercise, implementation of the methodology, and how results were reported and used. The following are key conclusions and recommendations:

### First, there is wide variation in the way costing methodologies are applied and terminology interpreted, particularly regarding common health system-related costs and capacity constraints, calling for more systematic guidance

Questionnaire responses revealed inconsistencies in implementation of costing methodologies and significant variations in interpretations of terminology and concepts. Teams from different backgrounds are likely to have different understanding of terms, make different assumptions, use different analyses, address different types of policy questions and seek different types of outputs. A shared understanding of terminology is essential to enable a comparison of costing exercises across different countries, including tools, methodologies and the use of results.

Online supplemental annex 3 provides some examples of how terminology could be presented in a reference guide for costing. The terms unit, per capita, incremental and indirect health system costs were included as they were found to be sources of confusion. For example, unit costs and per capita costs were often confused, in particular when estimating costs of population interventions. Importantly, despite its wide use in the DCP3 volumes,^20^ the authors encountered different uses of the term ‘health system costs’; in the survey, the term was changed to ‘indirect health system costs/overhead’.

10.1136/bmjgh-2022-010735.supp3 Supplementary data

While the choice of main methodology rightly depends on country needs, implementation of the same methodology should be consistent across exercises, which was not the case. For instance, some countries used a multiplier to account for overheads and others health system common costs; this is a major weakness because these costs are not linear (that is, they do not increase in the same proportion as service provision), they are fixed at the margin, and they are subject to threshold effects when new investment is necessary. Failing to properly incorporate these common ‘indirect health system’ costs has important consequences because the magnitude of these common costs is large, typically representing a major share of health services production function, including human resource and infrastructure-related costs.^21^ The potential for miscalculating the real cost of implementation is thus very significant.

Another area to highlight is the methodology for incorporating capital costs, including the costs and timing of infrastructure development. As mentioned above, only three countries included capital costs to reinforce the capacity of the health system in their long-run scenarios and only one country planned to integrate significant upgrades of the health system (online supplemental annex 2, table Q17). Yet, integrating investment plans with the costing of operational costs is needed if to be realistic when estimating funding needs.

### Second, estimating the costs of EPHS is not an exact science, so costing needs to integrate sensitivity analysis, acknowledge realistic ranges of uncertainty and be regularly updated

Uncertainties and data scarcity/quality issues create inaccuracies in costing estimates, thereby compromising confidence in their policy relevance. The cost of a good or service is the result of a particular production function and market circumstances at a specific point in time, so ex ante estimates are uncertain. While discussing the magnitude of estimated costs is beyond the scope of this paper, the range of cost estimates reported by the five countries is very large and differs significantly from the estimated costs of the model DCP3 packages.^22^

Large differences in total package costs and challenges related to understanding these differences highlight the need for full transparency in methodological approaches and assumptions, data sources and access to detailed cost results. EPHS cost estimates should ideally be presented with ranges of uncertainty. We recommend to systematically conduct sensitivity analysis and to report high and low estimates.

Finally, cost estimations were mostly carried out using ex ante projections instead of using cost data from interventions that are actually implemented (eg, through a pilot programme or in progressive implementation). Facilitating the use of ex post estimates would improve both validity and accuracy of future costing exercises and help better understand variation in package costs between countries over time, including how these costs can change with quality improvements and efficiency gains.

### Third, short-term one-off training is helpful but not sufficient: building long-term institutional capacity is needed for better reliability and policy relevance

Findings suggest that there is a critical need to reinforce the capacity of countries in costing EPHS, particularly in health economics and practical analytical skills to handle costing tools. At least half of the core costing teams did not have any prior skills in costing EPHS, and further training was not always provided during this exercise. The only country that did not receive any type of specific training placed the need for capacity building as highest priority to improve the process. High ratings for on-the-job training indicate that mixing skill levels in core teams is necessary, but it is not sufficient; institutionalisation and capacity strengthening in these areas within the MoH is critical. Indeed, most countries indicated they planned to update the costing exercise (online supplemental annex 2, table Q28). By repeating the process of costing the EPHS, countries can also build long-term local capacity and diminish their reliance on external experts. This supports local capacity building and helps to ensure that efforts on costing contribute to the wider policy making framework.

#### Fourth, costing and budgeting of EPHS are currently separate processes, hindering their implementation. Costing exercises need to be designed to support budgetary processes and to facilitate budget decisions.

The primary purpose of costing is to ensure that the EPHS can be implemented in a resource-constrained context. This goal is often confused with assessing efficiency (value for money), which is also important but mostly used in prioritisation—see Box S4 in Baltussen *et al*^23^—and done using different techniques.^24^ In order to fulfil its primary objective, costing needs to be designed so it can be used in the preparation of budgets. This calls for results being calculated and presented in ways that are compatible with budget processes so that they can be integrated into all steps of the budget cycle. The survey results show that most countries did not consider using the EPHS costing in budgeting. Investment plans were only done in one of the five countries and none provided a proposal for a mid-term budget framework. One country reported that, ex post, they would have changed the way they calculated and aggregated costs so that results could be used more readily in financing plans.

Integrating costing and budgeting implies that efforts be made to ensure that budgeting is considered as a purpose of costing ex ante. Beyond the need to reinforce capacity building, the participation of Ministry of Finance and budget officers in EPHS costing can ensure relevance and sustainability.

Costing that is conducted for the purpose of prioritisation and package design (as was the case in most countries) is often not sufficient to inform budgeting. In fact, decisions about prioritisation of interventions may ignore common costs, while they cannot be ignored in budgeting. Costing of EPHS for implementation purposes need to be linked to planning, budgeting, strategic purchasing and contracting needs. The literature reveals some directions for facilitating the integration of costing and budgeting.^25 26^ If budget impact is the main intended purpose of costing, the methodological choices and specific capacity building should be tailored towards this end goal. Additional guidance may be required on how to best structure total cost estimates so that they can be understood in terms of government budgets. More specifically, it is important to account for the national budget structure before designing the exercise. Budgets are organised differently in different contexts. Different approaches may be used. Budgets may be organised by line-item or through programme-based budgets, matching mission to expenditure.^26^ Budgets also usually separate investment and operational costs, or wage and non-wage expenditure. It is thus important to identify how the costing exercise relates to the country budget structure and the specific priority programmes identified by policy makers. When strategic purchasing agencies, such as National Health Insurance or National Health Service institutions are in place, EPHS costing also needs to translate into identifying a package that specifies the levels of public subsidy and co-payment.^27^ Finally, estimating budget impacts also implies knowing the incremental cost of the package relative to the status quo. The survey revealed that incremental costs were not systematically calculated and that the term was not uniformly understood. Analysis of incremental cost drivers can prove particularly useful in healthcare budgeting as well as for monitoring and evaluation.

## Conclusions

Given the limited resources in LLMICs, the costing of an EPHS is a critical step to ensure that the package is properly designed and integrated into a country’s budget perspective. In this paper, we examined how costing was carried out in five countries, how results were used, and what challenges they faced in carrying out the exercise. Despite the different contexts and the small number of countries considered (but all LLMICs and with access to similar resources), similar challenges were reported and stylised facts emerged.

We found that cost estimates could not always be used for intended purposes in policy making. More systematic guidance is needed to help teams align methodology with purpose and ensure that they find answers to specific methodological questions. Guidance and training are also necessary to build a common understanding of methods, concepts and terminology, so that they are applied consistently.

Costing an EPHS is challenging and results may be indicative rather than 100% accurate. To enhance confidence in results and promote their use in decision-making, we highlight the importance of calculating and reporting ranges of uncertainty, presenting sensitivity analysis/scenarios and being transparent in the methods and assumptions used.

The usefulness of costing is entirely dependent on the availability and accuracy of costing data. The study revealed that local data routinely collected by the MoH is the preferred source for costing teams. When it is not available, significant efforts need to be made to fill the gaps from multiple sources, including primary data collection. We recommend that costing data be routinely gathered and that local capacity be built to properly collect and use these data.

Lastly but importantly, the study emphasises the need to engage in long-term capacity building and to include costing as an integral part of institutionalising the process of designing an EPHS. Integration of costing teams within both policy making and budgeting structures will ensure that costing methods and processes are aligned with budgetary processes. This will also support the development of long-term local capacity for costing, a key challenge highlighted by all countries.

Insights gained from this study are limited by the number of countries that shared their experience. Although we believe that the challenges identified are representative of what other LLMICs can expect to encounter when carrying similar exercises, more robust conclusions and new insights could be obtained by reaching out to a larger number of countries. This will likely become possible as more countries are stepping up their efforts towards UHC. The questionnaire developed for this paper can be further improved and/or adapted to answer more specific questions, support future evaluations of costing methodologies and tools, extract lessons learnt and, by looking at changes in responses over time, learn how challenges are addressed. Beyond extended use of the questionnaire, recommended avenues for future research include developing best practices for sensitivity analyses in costing EPHS and developing the collection and use of cost data from activity monitoring. The latter is essential to analyse changes in EPHS costs over time, considering service quality and efficiency gains.

## Data Availability

All data processed from survey responses are uploaded as supplementary information (Annex 2).
