# Supplementary material for: Using costing to facilitate policy making towards Universal Health Coverage: findings and recommendations from country-level experiences
Source: BMJ Glob Health. 2023 Jan 18;8(Suppl 1):e010735. doi: 10.1136/bmjgh-2022-010735 (PMC9853124; doi:10.1136/bmjgh-2022-010735)
Supplement: Supplementary data [file bmjgh-2022-010735supp001.pdf]

DCP3 Disease Control Priorities

Country Translation Phase

Survey of Country Experiences – December 2021/January 2022

Questionnaire on Methodological Approaches and Tools for Estimating Costs and Budget Impacts

Preamble:

This survey is aimed at countries that engaged in costing exercises as part of the process to develop packages of essential health services with the view to reaching UHC-related Sustainable Development Goals by 2030.

Responses to the survey will be used to review and analyze country experiences on methodological approaches and tools for estimating costs and budget impacts. Responses will be considered as confidential. The report will not name individual respondents. Countries will not be identified when reporting on part II answers (normative feedback).

Terminology:

In the survey we use the term ***essential package of health services (EPHS)*** or simply ***package(s)*** to designate all UHC-compatible packages of health services that countries have developed. This is not indicative of how such packages are effectively called or should be called.

We use the term ***unit costs*** to mean the cost of providing a given health service (intervention) to one person. Note that the term is only used for interventions on the person.

We use the term ***per capita cost*** to mean total costs divided by total population (country or specific area of reference/catchment area)

We use the term ***indirect health system costs*** to mean all costs supported by the health system in relation to providing services but not directly related to the provision of that specific service (given capacity in place). This includes (but is not limited to) overhead costs.

Filling out the survey:

The survey may be filled collectively (multiple respondents with one single answer). It needs to be filled by people who were significantly involved in the costing exercise; this will enhance the relevance of responses.

If you have any reservations, clarifications, caveats, comments on a question, feel free to note these below each question/table. You may also contact:

Sylvestre Gaudin: slygaudin@gmail.com or **Wajeeha Raza:** raza.wajeeha@gmail.com

The survey has two parts:

Part I - Factual (objective) questions (Q1-28)

Part II - Normative assessment: personal feedback on experience (Q29-38)

DCP3 Disease Control Priorities
Country Translation Phase

COUNTRY: ______________________

CONTACT PERSON FOR THIS SURVEY: _______________________

Contact email: __________________________

I – Factual (objective) questions (Q1-29)

Q1. For what type of interventions did you carry out costing exercises? (Answer in Table Q1)

Instructions for Table Q1: Check the appropriate cell(s) with an X. Use last column to qualify your answer (for example if the exercise was not completed or completed only for specific types of interventions not others, etc.)

Table Q1					
Type	Description	Yes	No	Not done but planned	*optional: add text as needed*
A	Healthcare interventions (on the person)				
B	Population-based interventions				
C	Intersectoral interventions (involving sectors outside of health)				

Q2. Did you obtain a final estimate/range of estimates for the total cost/per capita cost of an essential package of health services (as defined on the front page) for your country? (Answer in Table Q2)

Instructions for Table Q2: Please, **provide the numeric values for this total cost/per capita cost**. Use columns 1 or/and 2 if you estimated the cost of a DCP3-inspired package adapted to the specific needs of the country and covering all essential health services for 80% of the population or more. If you estimated the costs of a package that is limited in scope (interventions covered) or coverage (less than 80%), please use column 3. Leave blank if you did not obtain (separate) estimates or if the exercise has not been completed.

Hint: several options are provided in column 1, use the line(s) that match what you already calculated (for example if you did not report the package of population interventions (B) separately from health care interventions (A), fill in the line A+B (leaving blank line A and B).

Currency/year: ____________ Check here (X) if estimates are in per capita terms: ____

Table Q2<br>Package type from Q1	Unconstrained country-specific version of DCP3 *EUHC* [(a)]<br>Estimated cost	Unconstrained country-specific version of DCP3 HPP[(a)]<br>Estimated cost	Package not based on DCP3 or limited in scope or coverage<br>**Please describe in a few words in same cell**<br>Estimated cost, content
A			
B			
A+B			
C			
A+B+C			

(a)The EUHC package includes 218 unique interventions, of which 13 population level interventions. The HPP is a sub-package of EUHC considered highest priority for low-income countries. The Intersectoral package in DCP3 includes 71

Gaudin S, *et al. BMJ Global Health* 2023; 8:e010735. doi: 10.1136/bmjgh-2022-010735

policies of which 29 were identified for early implementation. ***We are not asking here for the number of interventions in your package because what constitutes an intervention may be very different across countries*****.**

Q3. For each type of intervention A, B, C described in Q1, answer the questions in Table Q3 using your best estimate (or range).

Instructions for Table Q3: Indicate number of people who worked on each exercise even if these were the same people.

Table Q3	A *	B	C
When did the exercise begin (year)?			
How long did it take to complete the exercise (months)?			
Total number of people who directly participated in the costing exercise	N1=		
Total number of people who *indirectly* contributed[(a)]	N2=		

(a) People *indirectly* involved may include supervisors, expert reviewers, support staff. Include also in this category people who were not part of the costing team but worked on specific requests from the team such as providing intervention descriptions, population-in-need estimates, current budget numbers, etc.

**If the same people worked on A and B, indicate number of people who work on A and B in column A and check here (X):* ___

Optional for Q3: write here any caveat/information that you would consider important in order to correctly interpret the numbers you gave above: xx

Q4. Using categories in Table Q4, describe the people directly or indirectly involved in the exercise of costing the package of health interventions on the person (A) *[or A + B if not dissociated].* Define indirect involvement as above.

Table Q4	Number **directly** involved	Number or range **indirectly** involved
Total (from above)	N1	N2
Of which,<br>• Personnel from the ministry of health or other ministries/government agencies		
• People from local schools/universities		
• Other within country		
• International experts/external partners and universities		
• Other based abroad		

Q5. To the best of your knowledge, how did the local team acquire the skills needed to carry out the exercise? (Answer in Table Q5)

Instructions for Table Q5: Please, fill out all of column 1 with the number of people directly involved locally who benefitted from each source of training. If some types of training were more frequent or more substantial than others, use the second column to RANK how important the trainings were for the overall costing exercise (starting from 1 for most important and using same value number more than once if equally important)

Table Q5	Number of people in costing team	Ranking (optional)

p. 3/18

DCP3 Disease Control Priorities
Country Translation Phase

Table Q5	Number of people in costing team	Ranking (optional)
Pre-existing skills		
On the job training		
General technical support from development partners		
Specific training for the costing exercise:		
• locally		
• abroad		
General training courses just before or during the project timeline: (e.g. courses in health economics or other broad analytical skills)		
• Courses followed locally		
• Courses followed abroad		
Additional training requested by individuals in the team based on need		
Others? Specify: xx		

Q6. What was the primary purpose of the costing, and the intended use of the results (as expressed upfront prior to starting the costing work)? **Response is open (1-60 words ≈)**

Add text

Q7. What type of institutional or other country-specific context conditioned your methodological choices and the types of analyses you presented? (Answer in Table Q7)

Instructions for Table Q7: Please, check all that apply with an X. **Write XX if this type of context was determinant.** If the context only mattered for some types of interventions, specify in second column using A, B, C from Q1.

Table Q7	Check if considered X or XX	A, B, C? (optional)
Governance structure (including devolution if applicable)		
Public budgeting method (e.g. program-based, line-item, performance, etc.)		
Matching broad budget categories (operational/investment budget, Wage/non wage, etc)		
Specific planning methods (Health ministry and health facilities)		
National plans (content and timeline)		
Existing health financing schemes		
Current organization of the health system (referral for ex.)		
Importance/Role of private sector		
Country size, population, etc.		
Other? Specify xx		

Gaudin S, *et al. BMJ Global Health* 2023; 8:e010735. doi: 10.1136/bmjgh-2022-010735

Q8. What type of costing methodology did you use? (Answer in Table Q8)

Instructions for Table Q8: Write 1 for the main method used; write 2 if another method was used as a second choice on more than about 10% of interventions; write 3 if another method was used as a third choice or in few cases.

Table Q8 Used for (see Q1):	A<br>1-2- 3	B<br>1-2-3	C<br>1-2-3
**Bottom-up Ingredients-based approach**<br>i.e., from intervention unit cost* data Σ(q×p) to intervention cost (Σ(q×p) × expected utilization) to total package cost: specify the approach below			
• Normative approach<br>(ingredients are based on recommended protocol)			
• Real world activity-based approach<br>(ingredients are based on primary data collection for each intervention)			
**Top-down approach**; i.e., from aggregate expenditure data to intervention cost			
Survey of service providers			
Existing Disease Resource Group estimates			
Literature survey / contextualization of results from other costing exercises			
Other historical estimates – lump sum			
Expert opinion (no formal evidence given)			
Other? Describe: xx			

(Optional) If unsure about your answers, please summarize below in one sentence or/and formula how you obtained unit costs, cost of providing an intervention, and total package costs (see survey front page for terminology).

**(a)Unit costs** (as defined in terminology front page): xx<br><br>**(b)Total cost of providing the intervention**: xx<br><br>**(c) Total package cost**: xx

Q9. What tools did you consider/use for your costing exercises and for what purpose? (Answer in Table Q9)

Instructions for Table Q9: Check all that apply with X; add lines and describe the tool if other tools were used.

Table Q9	Considered		Used for	
	Yes	No or unknown	Estimating costs	Analyzing/ presenting results[(a)]
General:				
• One Health Tool (OHT)				
• Marginal Budgeting for Bottleneck Tool (MBBT)				
• Own spreadsheets from scratch (Excel or other)				
• Spreadsheets based on pre-existing templates (other costing exercise or other country)				

Table Q9	Considered		Used for	
	Yes	No or unknown	Estimating costs	Analyzing/ presenting results[(a)]
• Excel-based dynamic model				
• Microsoft Access / DEA-Solver-Pro				
Platform-specific tools				
• Core Revenue Analysis Tool (CORE-Plus tool)				
• Primary Health Care Costing Tool (PHC costing tool)				
• Community health planning and costing tool (CHPCT)				
• Other platform specific tool<br>Specify:				
Disease specific tools				
• SEEMS				
• TB service delivery costing tool				
• Other disease-specific tool<br>Specify:				
Analysis/presentation tools:				
• HIPtool				
• Optima Suite				
• Other analysis/ presentation tools<br>Specify: xx				
Other? specify: xx				

(a) i.e., aggregation, optimization, create tables and figures, etc.

Q10. What did you take into consideration when selecting **costing methodology and tools** (ex-ante)? (Answer in Table Q10)

Instructions for Table Q10: AFTER checking all considered in column 1 with an X, identify in column 2 the relative importance of those checked starting from 1 (most important). Use same number twice if equally important. Leave blank if not considered.

Table Q10	X if considered	Rank (1, 2, 3, etc.)
Time required to carry out the exercise		
Number of people needed		
Skills/expertise required - capacity development needs		
General guidance from international sources		
Availability/accessibility of specific guidance for the tool		
Prior knowledge of tools used		
Practical examples of other countries that carried out similar exercises		
Data issues (data requirements, data quality, data availability or access)		
Number of interventions to include in costing exercise		
Financial considerations		
Others? Specify: xx		

Q11. To assess the costs of health interventions on the person (A), what perspective(s) did you use?/ what types of costs were included? (Answer in Table Q11)

DCP3 Disease Control Priorities
Country Translation Phase

Instructions for Table Q11: Check appropriate cell with an X. Please, answer ALL lines, noting that items listed **are not mutually exclusive**.

Table Q11	YES	NO	In some cases (specify in few words within cell)
Economic perspective – i.e., resources required are valued not according to how much needs to be paid to get them but considering the value of their next best use			
Accounting/budget perspective (financial) – i.e., how much financing is needed to provide the intervention			
Provider perspective (health system)			
Patients perspective (demand-side)			
Societal perspective – i.e., including costs that accrue to all actors in society, including people who are neither providers nor recipients of the service.			
Inclusions/exclusions (regardless of perspective indicated above)			
▪ Currently donated resources were INCLUDED in costs, i.e. valued. (Check NO if donated resources were priced at 0)			
▪ Direct costs that are currently born by the user (health costs such as user fees, consumables, etc.)			
▪ patient-specific incidental costs (transportation, lost wages, day care, etc.)			
▪ Indirect health system costs/general overheads (not directly linked to activity level)			
▪ Investment costs to increase general capacity of the health system - Human capital, facilities, etc.			
Others? Specify: xx			

Q12. If you used an ingredient based approach, what cost components (inputs) were generally used to calculate costs? (Answer in Table Q12)

Instructions for Table Q12: Please, check all appropriate cells using an X (the list provided is not indicative of what "should" be included.). Check last column if the ingredient was included BUT not considered as a separate item. Note that some inputs listed are full services; Check these if you did not break these services into ingredients.

Table Q12.<br>Ingredient (input component)	YES	NO	Sometimes	NOT identified separately
Individual inputs				
Human Resources (personnel directly involved in providing the service)				
Drugs/Medicines				
Other materials and supplies/consumables				
Medical equipment				
Building use, vehicles, etc				
Management and supply chain costs				

Gaudin S, *et al. BMJ Global Health* 2023; 8:e010735. doi: 10.1136/bmjgh-2022-010735

DCP3 Disease Control Priorities
Country Translation Phase

Table Q12.<br>Ingredient (input component)	YES	NO	Sometimes	NOT identified separately
Overhead (administration, records, etc)				
Infrastructure costs (investment plan)				
Services (if not broken down by individual inputs)				
• Laboratory services				
• Radiology diagnostics services				
• Inpatient cost (catering ,hospitality services, etc)				
Other inputs or services not listed above (please list below; also note below if you used input adjustment parameters; add lines as necessary)				
xx				
xx				

Q13. Please provide a brief description below of how personnel costs were incorporated into intervention unit costs **if not based on input requirements from intervention descriptions**

Add text

Q14. Without going into details, how did you generally calculate and incorporate indirect health system costs into your estimates?

Add text

Q15. Please note below if you discounted any values, what values, and main assumptions used for discounting, if applicable. Also indicate here how you combined (or not) discount rates with amortization. Write **PASS** if the question is not obvious to you.

Add text

Q16. Did you include in total package costs any additional costs that you expect in relation to monitoring/implementation of the new package? If yes and if known, please indicate how much.

Add text

Q17. What is the time frame that best describes what you included in your cost estimates? Check all that apply with an X; you may also indicate corresponding time horizon in years. **If you did not consider any specific time frame, check here (X)**: ____

Table Q17	Yes	No	(optional) Corresponding Time horizon (years)
Short run<br>i.e., within existing capacity - no new investment			
Medium Run<br>i.e., considers additional equipment and capacity (including changes in health HR) within existing facilities			
Long run<br>i.e., considers possibility of significant upgrading and investment in new facilities			
If the items above do not fit the time horizon you considered, please give your own definition:			

DCP3 Disease Control Priorities
Country Translation Phase

Q18. DATA for Ingredient-based costing: What type of data did you use to calculate unit costs of interventions and from what source? (Table Q18)

Instructions for Table Q18: Use 1 for principal source of data used, 2 if second choice used sometimes, 3 if third choice used rarely)

Table Q18		Qs	Prices					Costed services
		Input requirements 1-2-3	Wages 1-2-3	Pharmacy 1-2-3	Equipment 1-2-3	Supplies/ Consumables 1-2-3	Other 1-2-3	Diagnostics/ radiology/ inpatient admissions/ surgery/ etc. 1-2-3
National / Local	Primary data collection – own survey of providers, facilities, etc.)							
	Data normally collected by Ministry of Health							
	Publically available local database							
	Existing cost studies from literature/reports							
	National Health Account data							
	Public expenditure data – include expenditure tracking/mapping resources							
	Commodity price lists – public sector							
	Commodity price lists – private sector							
	Data collected by local Non-Governmental Organizations							
	Other national/local							
International								
	WHO Compendium							
	WHO GHED							
	UN medicine price lists							
	International NGOs							
	Existing costing studies from literature/reports from other countries							
	Other International							

Q19. What type of data did you use for top-down costing method (if applicable)? In Table Q19, please check all that apply with an X.

Table Q19	From national/local sources	From international Sources	Indicate type of interventions for which this was used, if applicable.
National Health Accounts			
Budget appropriation data			
Public expenditure data – National			
Public Expenditure data – Provincial/Local			
Specific expenditure Mapping/tracking resources			
Others? Specify: xx			

DCP3 Disease Control Priorities
Country Translation Phase

Q20. What type of data did you use to estimate demand for the intervention (population in need, coverage/utilization) and from what source? If another team provided the estimates, please indicate here.

Add text

Q21. What guided your normative choice of target population coverage? In Table Q21, check all that apply with an X. Note that items suggested are in no specific order and may be overlapping.

Table Q21	Yes	No	Check if principal source(s)
National Strategic Plan/National programs for specific diseases			
International targets/commitments (such as UHC)			
Deliberations with engaged stakeholders – Local program leads and other in country			
Deliberations with engaged stakeholders – Development partners and other international			
Current infrastructure/Human resource capacity			
Investment plan			
Feasibility			
Others? Specify: xx			

Q22. In planning for implementation, budgeting, or running any scenario involving time, did you consider/ make assumptions about expected changes over time? In Table Q22, please, check all that apply with an X in column 1 and briefly specify main assumptions, if any, in column 2.

Table Q22	Considered	Assumption(s) made, if any
Expansion of coverage [(a)]		
Changes in expected demand		
Changes in wages		
Changes in Pharmaceutical prices		
Changes in health system capacity		
Changes in other prices		
Technological changes		
Macroeconomic conditions (inflation, growth, employment)		
Others? Specify: xx		

(a) Include scenarios you may have run for progressive expansion of the package, with increasing coverage or increasing number of health interventions in future years.

DCP3 Disease Control Priorities
Country Translation Phase

Q23. Outputs of the exercise: what type of measures did you calculate/present for healthcare interventions (A) and for population interventions (B)? In Table Q23, check all that apply with an X.

Table Q23 Reported for (see Q1):	A	B
Average/typical unit costs of an intervention (per user/year or other similar unit)		
Total cost by intervention (Unit cost x estimated demand)		
Per capita cost by intervention (Total cost of the intervention divided by total population of the country or other geographical catchment area) [(a)]		
Total cost of packages relative to "do nothing"		
Incremental costs (total or per capita cost of delivering the package relative to status quo)		
Per capita cost for packages or sub-packages[(a)]		
Cost drivers (%)		
Costs by type of input (for example to fit specific budget configurations or for planning purposes)		
Costs by program categories (to fit budget program)		
Indirect health system costs/overheads		
Investment versus operational costs		
Others? Specify: xx		

(a) Note: per capita costs are generally used so that figures can be compared to other countries/areas

Q24. What categories were used to report costing results (in output reports or any other presentation)? (Answer in Table Q24)

Instructions for Table Q24: Please, check all that apply with an X. Some "by" categories may not be applicable for some measure listed, or not appropriate for your specific context, you may leave blank or write N/A. Definitions for terms marked with an * are provided in the terminology section on the front page

Table Q24	For each Inter-vention	By disease category	By level of healthcare (Primary/ sec., etc)	By health delivery platform/ health facilities	By type of healthcare provider/ HR	By location (geogr.)	For National aggregates
Unit costs*							
Average unit costs							
Total cost							
Per capita costs*							
Indirect health-system costs*							
Cost drivers							
Specific Input requirements[(a)]							
**If other "by" categories were used for any reported measure, please specify**: xx							

(a) Input requirements are expressed in quantities (not monetary terms), for example one may want to use your estimates of total number and type of personnel required at a given platform level, number of beds, quantity of medicines, etc. to be used for planning purposes.

DCP3 Disease Control Priorities
Country Translation Phase

Q25. Did you carry out sensitivity analysis (to assumptions made, uncertain data, etc.) or consider different scenarios (based on varying conditions, timelines, investment plans, applicability to different areas, etc)? Check with an X all that apply in Table Q25.

Table Q25	Yes	No	Specify variables/assumptions considered if applicable (optional)
Sensitivity to different data specification (e.g., using different sources for prices or recommended protocols, using average *vs.* marginal prices, etc.)			
Sensitivity to different assumptions (e.g., discount rates, amortization, available technology, linear/non linear relationships, etc)			
Other sensitivity analysis			
Different scenarios on human resources for health			
Different scenarios on investment plans			
Different scenarios on extent of coverage			
Different geographical expansions			
Others? Specify: xx			

Q26. Were any of your cost results compared to existing ones? (Answer in Table Q26)

Instructions for Table Q26: Please write **0 if NOT done, 1 if done, 2 if done partially** (on some costs or some sub-categories). If comparisons were not formally reported but you know the issue was raised in discussions or presentation, write "discussed".

Table Q26 - Own results compared to:	DCP3 0-1-2	Literature 0-1-2	Other countries 0-1-2	Other in-country 0-1-2	*Other? (specify)* 0-1-2
Total or per capita costs of packages					
Unit costs of individual interventions					
Total costs by intervention					
Total costs by platform					
Total costs by disease or program area					
Cost of population-level interventions					
Costs of intersectoral interventions					
Others? Specify: xx					

Other sources of comparison if any: xxx

If you answered 0 in first column, please indicate here if you were aware that DCP3 recommended packages of essential interventions when you did the costing exercise **YES/NO**: ____ , and that DCP3 reported cost estimates for their EUHC and HPP packages: **YES/NO**: ____

DCP3 Disease Control Priorities
Country Translation Phase

Q27. To the best of your knowledge, what types of decisions were facilitated by the results of your costing exercise? In Table Q27, please check all applicable with an X. Write XX if you think the exercise was determinant.

Table Q27<br>Type of decision<br>(order is not indicative of importance)	Facilitated by your costing exercise<br>X or XX	Not yet used for this intent but planned to be used<br>X
Prioritization of interventions		
Annual budgeting – National/Local		
Long-term budgeting – National/Local		
Implementation scenarios (packages)		
Advisory committee meetings		
Implementation at different levels		
Service delivery planning		
Health workforce planning		
Medicines, equipment, supplies procurement		
Contracting-out (PPPs, NGOs, etc.)		
Business case/Resource gaps for funders		
Infrastructure Investment plans		
Quality evaluation of current services		
Others? Specify: xx		

Q28. Do you (your country) have plans to revise/update the costs of EPHS? **Yes/No** ____

If yes above, indicate whether periodic revisions are planned and how often you expect to revise (give frequency in years): xxx

Thank you for answering part I, please, continue to normative assessment (part II)

Gaudin S, *et al. BMJ Global Health* 2023; 8:e010735. doi: 10.1136/bmjgh-2022-010735

DCP3 Disease Control Priorities
Country Translation Phase

II- Normative assessment: personal feedback on experience (Q30-39)

Q29. Did you find sufficient guidance upfront on the features of costing methodologies and costing tools to make informed decisions on the most suitable ones for your intended purposes? In table Q29, rate from 0 if you found no guidance to 10 if guidance was largely accessible/sufficient for your purpose.

Table Q29	To select appropriate methodology Rating 0-10	To select appropriate tools Rating 0-10
General accessibility of guidance		
General usefulness of guidance		
Specific features:		
• Skills/expertise required		
• Time required		
• Data required		
• Type of output produced relative to anticipated needs		
• Feasibility of different types of analyses		
• Other? Specify: xx		

Q30. Ex-post, what do you think should be most important to take into account when selecting methodologies and tools. In Table Q30, please rate from 0 to 10, 10 being most important.

Table Q30	Rating 0-10
Time required to carry out the exercise	
Number of people needed	
Skills/expertise required - capacity development needs	
General guidance from international sources	
Availability/accessibility of specific guidance for the tool	
Prior knowledge of tools used	
Practical examples of other countries that carried out similar exercises	
Data issues (data requirements, data quality, data availability or access)	
Number of interventions to include in costing exercise	
Financial considerations	
Others? Specify: xx	

Gaudin S, *et al. BMJ Global Health* 2023; 8:e010735. doi: 10.1136/bmjgh-2022-010735

DCP3 Disease Control Priorities
Country Translation Phase

Q31. Reflecting back on the TOOL(S) you used, what tool and, if appropriate, what specific feature or characteristic of the tool did you find most useful in producing and analyzing/presenting the cost estimates? In Table Q31, fill in the tool and specific feature you found most useful, and check for what use(s).

Table Q31		To produce cost estimate	To analyze and present [(a)] cost estimates
Main tool (add lines as necessary)	Specific feature if any	X	X

(a) e.g. aggregation, optimization, creating scenarios, sensitivity analysis, creating tables and graphs, etc

Q32. Costing results need to provide reasonable expectation on magnitude so they can be useful in decision-making. Please rate below your appreciation of how the costing exercise you carried out fulfilled this goal (0- Results judged not good enough to inform policy; up to 10, results were trusted as sufficiently reliable to be used in decision making)

Note that the rating is NOT about the level of accuracy of results or anticipated margins of error, just about how they were perceived as good enough for decision making.

Rating (0-10): ____

Q33. In general, please rate how useful you think costing estimates/analyses are or can potentially be in different categories of decision-making. Write N/A or leave blank if you have no opinion/don't know.

Table Q33<br>(Items listed below in no specific order)	This costing exercise<br>Rating 0-10	Potentially<br>Rating 0-10
Annual budgeting – national/local		
Long-term budgeting – national/local		
Planning implementation scenarios		
Prioritization of health interventions		
Advisory committee meetings		
Implementation at different levels		
Service delivery planning		
Health workforce planning		
Medicines, equipment, supplies procurement		
Contracting-out (PPPs, NGOs, etc.)		
Business case/Resource gaps for funders		
Infrastructure investment plans		
Quality evaluation of current services		
Demand management (through shadow pricing)		
Others? Specify: xx		

DCP3 Disease Control Priorities
Country Translation Phase

Q34. How do you perceive the value of different types of capacity building to carry out costing exercises? For each listed in Table Q34, give a score from 1 to 10 to gauge how you perceive the value of the skills acquired in terms of facilitating the exercise (based on completed exercise) and ensuring adequate continuity (value for future exercises).

Table Q34	Perceive value for this exercise<br>Rating 0-10	Perceived value for future exercises<br>Rating 0-10
On the job training		
General technical support from development partners		
Specific training for the exercise:		
• Classes or workshops organized locally		
• Classes or workshops organized abroad		
General training courses<br>(for ex. courses in health economics or other broad analytical skills).		
• Courses followed locally		
• Courses followed abroad		
Additional training requested by individuals in the team based on need		
Other? Specify xx		
**Please add below any type of capacity building that you would suggest (not listed above or if you wish to be more specific):** xx		

Q35. In your view, EX-POST, what was (were) the challenge(s) you faced in carrying out the costing exercise (including building estimates and analysis)? Rate from 0 to 10, 10 being the biggest challenge.

Table Q35 - Challenges (some may be overlapping)	Rating 0-10
Time required to carry out the exercise	
Number of people needed to carry out the exercise	
Skills/expertise required to carry out the exercise	
Availability or timeliness of capacity training	
Availability/accessibility of specific guidance for costing tools	
Lack of practical examples from other countries	
Data issues (data requirements, data quality, data availability)	
Lack of information on existing health system capacity/investment needs	
Treatment of investment costs	
Understanding of terminology	
Coordination/timing issues, including with other teams	
Difficulty in explaining results to decision makers	
Covid-19 related issues	
Specific country context (specify below)<br>xx	
Others? Specify: xx	
If you wish, please give details below regarding the mains issues you faced: xx	

Gaudin S, *et al. BMJ Global Health* 2023; 8:e010735. doi: 10.1136/bmjgh-2022-010735

DCP3 Disease Control Priorities
Country Translation Phase

Q36. Ex-post and in consideration of how results were affected by specific methods used as well as how results were used, could the costing exercise be **simplified** without making much of a difference? **Yes-No**: ☐ **If yes, what could be simplified/ignored in your view**?

add text - open

Q37. If you were asked to redo the exercise today, what would you advise to do differently to make the costing results more useful in decision making?

Add text - Open

Q38. OPEN QUESTION: Please add any information you think necessary here so the review can best benefit countries in the development of their own UHC-compatible health benefit package

Add text – Open

THANK YOU FOR YOUR CONTRIBUTION

Please tell us approximately how long it took to respond to this survey: xx
