# Supplementary material for: Using costing to facilitate policy making towards Universal Health Coverage: findings and recommendations from country-level experiences
Source: BMJ Glob Health. 2023 Jan 18;8(Suppl 1):e010735. doi: 10.1136/bmjgh-2022-010735 (PMC9853124; doi:10.1136/bmjgh-2022-010735)
Supplement: Supplementary data [file bmjgh-2022-010735supp002.pdf]

SUPPLEMENT - ANNEX 2 (SA2): SUMMARY OF SURVEY RESULTS – ALL QUESTIONS

Table A2.1. Mapping of survey questions by themes

TOPIC	Factual Information	Normative Feedback
COSTING EXERCISE: SET-UP AND FOLLOW-UP	Q1; Q3; Q28	
CONTEXT	Q7	
PEOPLE AND SKILLS	Q4-5	Q34
METHODS AND TOOLS	Q8-17; Q21	Q29-31
DATA	Q18-20	
ANALYSIS AND OUTPUTS	Q2, Q22-26	
PURPOSES AND USES OF OUTPUTS	Q6, Q27	Q32-33
CHALLENGES/LESSONS LEARNED		Q35-38

The next tables are labelled using the corresponding survey question(s) and ordered as in the survey

Italics are used if the text is a direct quote from responses.

Table Q1. Scope

Category of intervention:	Individual based healthcare	Population-based	Inter-sectoral
Afghanistan	Yes	Yes	No
Ethiopia	Yes	Yes	Nutrition only (a)
Pakistan	Yes	Yes	No
Somalia	Yes	Yes	No(b)
Sudan	Yes	Yes	No

(a) Nutrition is under the responsibility of the MoH in Ethiopia (see reference [9] in the paper). Other countries may have included nutrition but did not consider intersectoral.
(b) Explanatory text included: "*Intersectoral interventions were included but not costed; MoH to promote action by the line ministers expected to finance these interventions.*"

Table Q2. Total estimated per capita costs of EPHS in the five countries

Country	**Range of reported cost estimates in 2020 USD per capita/year [(a)]**	**Type of EPHS[(b)]**
Afghanistan	6.5	Primary care package based on DCP3 HPP, 80% coverage – no other estimate was given.
Ethiopia	40-94	The figure of USD 40/capita corresponds to a "business as usual" scenario (Hailu *et al*, 2021). The high figure is reported for 80% coverage in 2030 but not based on HPP or E-UHC – A middle scenario estimated at USD 69/capita corresponds most closely to reaching SDG by 2030
Pakistan	13-72	The low figure is for immediate implementation, district level services (mostly primary care)/current coverage. Progressive implementation of the district level package planned till 2030 up to USD 54/capita. The high figure is the full desired package at 80% coverage, based on DCP3 E-UHC, prioritized for Pakistan.
Somalia	7.8 to 33	Based on HPP, 80% coverage, the low figure is for the reduced package for immediate implementation, the high figure is for the primary care package planned for 2030
Sudan	23.3-98	The low figure is for an immediate implementation package (details were not given); the high figure is based on WHO EMRO guidance adapted and extended by local clinical expert panels (all platforms and full population coverage).

(a) Values reported in the surveys were all translated to 2020 USD. When per capita cost was not provided, it was calculated using 2020 population estimate (Sudan).
(b) The term EPHS is used as in the paper but may not be the term used by the country. All the respondents indicated they were aware of the DCP-3 recommended packages of interventions toward UHC (the E-UHC and HPP, as defined in references [5,22]), as well as their cost estimates (Q26). Details of packages in paper references [4-9].

Table Q3. Timing and Team Sizes

	Afghanistan	Ethiopia	Pakistan	Somalia	Sudan
Year exercise started	2019	2018	2018	2019	2020
Costing exercise completed in (months)	6	6-18[(a)]	12	20	12
Number of people in the team (direct involvement)	2	8	5	8	12
Number of people assisting the team (indirect involvement)	3	30	5	16	11

(a) The second number includes time taken for the revision process.

Gaudin *et al*. – DCP Country-level experiences on costing EPHS – SA2

Table Q4. Team composition

Costing Team	Mean	Afghanistan	Ethiopia	Pakistan	Somalia	Sudan
DIRECT – number of people	***7***	2	8	5	8	12
Of which:						
MoH or other Gov	***62%***	100%	50%	20%	50%	92%
Local schools/universities	***12%***	0%	0%	60%	0%	0%
Other within country	***4%***	0%	13%	0%	0%	8%
International experts/external partners and academia	***22%***	0%	38%	20%	50%	0%
INDIRECT – number of people	***13***	3	30	5	16	11
Of which:						
Ministry of Health/Gov	***47%***	67%	50%	40%	25%	55%
Local schools/ universities	***13%***	0%	17%	40%	6%	0%
Other within country	***7%***	0%	17%	0%	19%	0%
International experts (external partners and academia)	***33%***	33%	17%	20%	50%	45%

Table Q5. Skills and capacity building

	Mean	Afghanistan	Ethiopia	Pakistan	Somalia	Sudan
% with pre-existing skills	***39%***	50%	38%	40%	50%	17%
% who learned “on the job”	***48%***	50%	0%	100%	50%	42%
Capacity building other than learning by doing/on-the-job training						
Volume (Σ of beneficiaries over all type of training)	***9.4***	7	5	9	0	26
Volume/team size	***1.39***	3.50	0.375	1.80	0	1.25
Capacity building by type						
On the job training	***33%***	13%	0%	36%	100%	16%
General technical support from development partners	***25%***	25%	60%	21%	0%	16%
Specific training for costing:						
Locally	***21%***	50%	0%	21%	0%	32%
Abroad	***7%***	13%	0%	21%	0%	0%
Relevant general training courses						
Locally	***13%***	0%	40%	0%	0%	23%
Abroad	***0%***	0%	0%	0%	0%	0%
Additional training requested by individuals in the team based on need	***3%***	0%	0%	0%	0%	13%

Table Q6. Primary purposes of EPHS costing

Country	Full open-ended response	Identified purposes
Afghanistan	*The objective of the costing exercises was to identify the estimates of individual services costs, individual program cost, individual health facility total cost as well as the total cost of the Integrated Package of Essential Health Services (IPEHS) aiming to inform efficiency and support contracting for the IPEHS services, demonstrating value for money, planning the allocation of resources and advocating for government and donor financing.*	• Support efficiency<br>• Demonstrating value for money<br>• Planning resource allocation<br>• Contracting<br>• Resource mobilization
Ethiopia	*(a) to understand the required resource need for implementation of the package (b) to revisit the package according to potentially resource need assumption (c) to understand the required addition resource, and mobile more resource*	• Resource needs/Affordability<br>• Reprioritization based on resource constraint<br>• Resource mobilization
Pakistan	*The costing exercise was meant to support deliberation by providing an indication of what interventions the country can afford in their resource envelope*	• Resource needs/Affordability<br>• Reprioritization based on resource constraint
Somalia	*The government wanted to know the cost of the package in order to secure adequate funding from local and international sources, the latter being the main financing source for the Somalia EPHS. The government was also convinced that a costed package will help raise funds from domestic and international sources by providing evidence of what results could be achieved if the budget requested were made available to the health sector to deliver the package.*	• Resource mobilization<br>• Demonstrating value for money
Sudan	*To develop costing at 2020 prices of health benefit package candidate interventions. Estimates included costs of current coverage and costs of achieving full population coverage for each intervention. The costing are to be used to support an assessment of affordability of priorities identified for inclusion in the health benefits package.*	• Resource needs/Affordability<br>• Reprioritization based on resource constraints

Table Q7. Role of country context in choice of methods/tools/analyses presented

**Country context**[(a)]<br>**Ranked by average score**[(b)]	**Countries Influenced by the category**	**Of which, number where determinant**	**Average score** [(b)]
Specific planning methods (Health ministry and health facilities)	4	1	1
Governance structure (including devolution if applicable)	4	0	0.8
Matching broad budget categories (operational/investment budget, Wage/non-wage, etc)	3	1	0.8
Existing health financing schemes	3	1	0.8
Importance/Role of private sector	3	1	0.8
Current organization of the health system (referral for ex.)	3	0	0.6
Country size, population, etc.	3	0	0.6
Public budgeting method (e.g., program-based, line-item, performance, etc.)	2	0	0.4
National plans (content and timeline)	2	0	0.4

(a) One country added donor financing (negotiations with partners) as a determinant factor in the choice of methodology and tools. This item had not been included as a country context options in the survey.

(b) We use values of zero the choice was not influenced by a given topic, 1 if it was influenced and 2 if it was determinant. A maximum score of 2 would be obtained if all countries had marked the category as determinant

Table Q8. Costing methodologies

N=Number of countries	Main method N	Secondary method N	Occasionally used N	Average score [(a)]
Main methodology used (sorted from most to least used based on average score) [(a) (b)]				
Bottom-up Ingredients-based , normative (based on best practice - protocols)	4	1	0	2.6
Top-down approach	0	3	1	0.7
Bottom-up Ingredients- based on real world activity (primary data collection)	2	0	0	1.2
Literature survey / contextualization of results from other costing exercises	0	2	1	0.5
Expert opinion (no formal evidence given)	0	2	0	0.4
Survey of service providers	0	1	0	0.2
Existing Disease Resource Group estimates	0	1	0	0.2
Other historical estimates – lump sum	0	1	0	0.2
Specific methodology used for population-based interventions [(c)]				
Top-down approach	2	1	0	1.4
Bottom-up Ingredients-based - normative (protocol)	2	0	0	1.2
Bottom-up Ingredients-based - real world activity	1	1	0	0.8
Survey of service providers	0	1	1	0.3
Existing Disease Resource Group estimates	0	1	0	0.2
Literature survey / contextualization of results from other costing exercises	0	1	0	0.2
Other historical estimates – lump sum	0	1	0	0.2
Expert opinion (no formal evidence given)	0	1	0	0.2

(a) The score is calculated using a 3 for the main method, 1 for a secondary method and 0.5 when used occasionally
(b) Two countries reported the main method used in the costing exercise without distinguishing between platform-based healthcare services (interventions on the person) and population-based services.

Table Q9. Costing tools

**N=Number of countries**<br><br>**Tools sorted from most used (by category)**	**Used for estimating costs N**	**Used for analysis and presentation N**	**Tool not considered N**
**General tools**			
Own spreadsheets from scratch (Excel or other)	3	4	1
One Health Tool (OHT)	3	1	1
Excel-based dynamic model	1	0	4
Marginal Budgeting for Bottleneck Tool (MBBT)	0	0	5
Spreadsheets based on pre-existing templates (from other costing exercises or other country)	0	0	5
Microsoft Access / DEA-Solver-Pro	0	0	5
**Platform-specific tool**			
Core Revenue Analysis Tool (CORE-Plus tool)	1	0	4
Primary Health Care Costing Tool (PHC costing tool)	0	0	5
Community health planning and costing tool (CHPCT)	0	0	5
Other platform specific tool	0	0	5
**Disease specific tools**			
SEEMS	0	0	5
TB service delivery costing tool	0	0	5
Other disease-specific tool	0	0	5
**Analysis/presentation tools:**			
HIPtool	0	1	4
Optima Suite	0	0	5
Other analysis/ presentation tools	1	1	4

Table Q10. Considerations in the selection of methodology and tools

**N=Number of countries**<br>**Ordered by score** [(a)]	**Considered**<br>**N**	**Top rank**<br>**N**[(a)]	**Average rank**<br>[(a)(b)]
Skills/expertise required - capacity development needs	5	3	1.5
Availability/accessibility of specific guidance for the tool	5	2	1.5
Time required to carry out the exercise	5	2	2.25
Data issues (data requirements, data quality, data availability or access)	5	2	2.75
Prior knowledge of tools used	5	2	3.5
Number of people needed	5	1	1.75
General guidance from international sources	5	1	3.75
Practical examples of other countries that carried out similar exercises	4	1	4.5
Financial considerations	2	1	7
Number of interventions to include in costing exercise	3	0	6

(a) One country did not provide a ranking of considerations.
(b) Range is from 1 to 9. Countries could give the same rank to different considerations. One country ranked all they considered as equally important and only one country ranked importance from 1 to 8. When something was not considered, and therefore not ranked, a 9 was assigned to calculate the mean rank.

Table Q11. Costing perspectives and specific inclusion/exclusions

**Perspectives (multiple choice possible)**	**Number of countries N**
Economic perspective	2
Accounting/budget perspective (financial)	4
Provider perspective (health system)	4
Patients perspective (demand-side)	0
Societal perspective	0
**Combinations (the total adds to 5 countries)**	
Accounting/budget perspective (financial) - no other approaches checked	1
Financial + provider perspectives	2
Economic + provider perspectives	1
Economic + Accounting/budget + Provider	1
**Inclusions/exclusions (regardless of perspective indicated above)**	Included [(a)]
Currently donated resources (non-zero value)	4 (5)
Direct health costs currently born by the user (user fees, consumables, etc.)	2
patient-specific incidental costs (transportation, lost wages, day care, etc.)	0
Indirect health system costs/general overheads (not directly linked to activity level)	4 (5)
Investment costs to increase general capacity of the health system (HR, facilities, etc.	3

(a) The number of countries that INCLUDED a given type of costs are indicated in the first number; the number of in parentheses includes countries that indicated including these costs only in specific circumstances)

Table Q12. Ingredients used in the estimation of intervention costs

	**Number of countries** [(a)]
**Primary inputs:**	
· Human Resources (personnel directly involved in providing the service)	5
· Drugs/Medicines	5
· Other materials and supplies/consumables	5
· Medical equipment	5
· Building use, vehicles, etc	3
**Services (if not broken down by primary inputs):**	
· Laboratory services	3 (4)
· Radiology diagnostics services	4 (4)
· Inpatient cost (catering ,hospitality services, etc)	5 (4)
**Others costs (not broken down by primary inputs)**	
· Management and supply chain costs	3
· Overhead (administration, records, etc)	4
· Infrastructure costs (investment plan)	2

(a) The number of countries that INCLUDED a given type of costs are indicated in the first number; The number in parentheses includes countries that indicated including these costs only in specific circumstances

Q13-16. Open-ended questions on implementation of methodology

	Country	Open-ended response
Q13. Personnel Costs (a)	Afghanistan	*Minutes of time allocated by health workers in order to provide services were estimated based on expert opinion and direct observation.*
	Ethiopia[(a)]	*Based on input requirements from intervention descriptions*
	Pakistan	*Based on input requirements from intervention descriptions*
	Somalia	*Personnel costs were included in the intervention description but the calculation of the unit cost, the HR, infrastructure, logistics and program cost were calculated based on the health system input* [(b)]
	Sudan	*Based on One Health Tool and recommendations from WHO EMRO intervention list.*
Q14. Indirect health-system costs	Afghanistan	*The tool has the capacity to allocate the indirect cost for each service*
	Ethiopia	*Left Blank*
	Pakistan	*We added a 1.6 multiplier to incorporate any additional health system costs (same for all interventions)*
	Somalia	*We considered indirect cost as part of the health system support need which include in service training of staff, supervision, coordination and the work of the regional and district management teams.*<br>*From Q8: The overhead included are those of the sub-national level. They include private service providers that may contracted by the government.*
	Sudan	*Based on OHT*
Q15. Discounting	Afghanistan	*Values were not discounted.*
	Ethiopia	*All costs discounted at 6%*
	Pakistan	*Discounting was used as part of the formula to determine the cost of using the equipment. We used a 3% discount rate to account for depreciation. This was applied on top of the amortization rate based on equipment lifetime.*
	Somalia	*We used the discount impended in the system which , I think was 3%*
	Sudan	*All done in 2020 prices - no discounting*
Q16. Inclusion of M&E and other implementation costs	Afghanistan	*We included M&E costs for the new interventions proposed in the IPEHS package. The difference in M&E between the previous package and the new package was estimated and reported.*
	Ethiopia	*Yes, M&E costs, no value*
	Pakistan	*No, but this should be included in the health system cost*
	Somalia	*No*
	Sudan	*No*

[(a)]Respondents were asked to answer the question if personnel costs were not incorporated based on input requirements from intervention description sheets.
[(b)] Original answer for Somalia was unclear. The reported answer is from a follow-up clarification by email and could not be clarified further.

Table Q17. Time perspective

Time horizon	Described in survey as	Number of countries
Short run	Within existing capacity - no new investment	2
Medium -run	Considers additional equipment and capacity within existing facilities	3
Long-run	Considers possibility of significant upgrading and investment in new facilities	1

Table Q18. Data sources used to calculate unit costs, from mostly used to least used

**Based on responses from 4 countries (a)**	***Index Score (b)***	**Input requirements (c)**	**Wages (c)**	**Drug prices (c)**	**Equip. Price (c)**	**Supplies, consum. & other prices (c)**	**Costed services (c)**
Sources sorted by Index score (b)							
Data normally collected by Ministry of Health	***0.71***	3 (4)	4	4	3 (4)	3 (4)	3 (4)
Public expenditure data	***0.41***	2	2	2	2	2	2
Primary data collection – own survey of providers, facilities, etc.	***0.33***	2	1 (2)	2	1 (2)	1 (2)	1 (2)
Publicly available local database	***0.18***	1	1	1	1	1	1
Existing cost studies from literature/reports - National/local	***0.16***	0 (1)	0 (1)	0 (1)	0 (1)	0 (2)	0 (1)
Commodity price lists – public sector	***0.14***	0	1	1	1	1	1
Existing costing studies from literature/reports from other countries	***0.10***	1 (2)	0	0	0	0 (1)	0
Data collected by local Non-Governmental Organizations	***0.09***	1	0 (1)	0 (1)	0 (1)	0 (1)	0 (1)
National Health Account data	***0.05***	1	0 (1)	0	0	0	0
WHO GHED	***0.04***	1	0	0	0	0	0
UN medicine price lists	***0.04***	1	0	0	0	0	0
International NGOs	***0.04***	1	0	0	0	0	0
Commodity price lists – private sector	***0.01***	0	0	0 (1)	0 (1)	0 (1)	0 (1)
Other national/local	***0.00***	0	0	0	0	0	0
WHO Compendium	***0.00***	0	0	0	0	0	0
Other International	***0.00***	0	0	0	0	0	0
**National/Local vs. International Sources**							
Countries that used any type of National/Local data as primary source		ALL	ALL	ALL	ALL	ALL	ALL
Countries that used any kind of international data as primary source (d)		Somalia	NONE	NONE	NONE	NONE	NONE

(a) The data for one country could not be used because all cells were indiscriminately checked. In the absence of clarification, we dropped the data. The above table is therefore based on responses from 4 countries

(b) The index score is calculated by attributing a value of 3 when the data was marked a principal use, 1.5 is secondary usage and 0.5 if rarely used. The different types of data needs (6 columns) were aggregated. The total was normalized using the maximum possible score of 80

(c) Number of countries that relied primarily on the data source. The number in parentheses adds countries that used this type of data as secondary source or occasionally.

(d) Countries did not report on sources of data that they did not collect, in particular, when they used default values from tools such as OHT. The use of international data is therefore underestimated.

Table Q19. Data sources for top-down costing methodology

Country	Data sources
Afghanistan	National-level Public Expenditure and budget data
Pakistan	Provincial/Local level expenditure data and expenditure tracking
Sudan	NHA + some national budget data for specific disease programs
Ethiopia and Somalia	Top-down method was never used

Table Q20. Data sources used to estimate target populations (expected demand by intervention)

Source	Type of data	Number of countries [(a)]
Health management information system (HMIS)	Utilization	3
Global BoD data (Not reported whether IHME or WHO)	Disease Prevalence/incidence	2
Demographic and Health Survey (DHS)	Utilization and disease prevalence/incidence	2
Other national surveys	Various complements	2
Data from STEPS survey (STEPwise Approach to NCD Risk Factor Surveillance, WHO)	NCDs risk factors	1
Annual performance reports and other national	Utilization and other complementary data	1
Literature and Expert clinical advice (b)	ALL (b)	1
Default assumptions (c)	In case other data is not available	1

(a) Countries described the data sources for target population I words. N is the number of countries who included a given source in their answer.
(b) One participation country indicated “literature and expert advice” as the sole source of data to estimate target population. Expert advice was likely based on some of the standard sources listed above.
(c) One country assumed current coverage (utilization) at 10 percent of the population when other data was not available.

Table Q21. Normative choice of target population coverage: sources of guidance

N=Number of countries<br>Source of guidance ranked by score [(a)]	N where source used	N where determinant	Average score [(a)]
International targets/commitments (such as UHC)	5	1	1.2
National Strategic Plan/National programs for specific diseases	4	1	1
Deliberations with engaged stakeholders – Local program leads and other in country	4	1	1
Current infrastructure/Human resource capacity	3	1	0.8
Investment plan	2	1	0.6
Feasibility	3	0	0.6
Deliberations with engaged stakeholders – Development partners and other international	2	0	0.4

(a) We use values of zero the choice was not influenced by a given topic, 1 if it was influenced and 2 if it was determinant. A maximum score of 2 would be obtained if all countries had marked the category as determinant.

Table Q22. Consideration of expected changes over time

Categories listed in the 3 countries that considered expected changes over time. Ranked from most cited	Number of countries
Expansion of coverage [(a)]	2
Changes in expected demand	2
Changes in health system capacity	2
Macroeconomic conditions (inflation, growth, employment)	2
Changes in wages	1
Technological changes	1
Changes in Pharmaceutical prices	0
Changes in other prices	0

(a) Two of the five countries did not consider any kind of change over time
(b) This category includes scenarios run for progressive expansion of the package, with increasing coverage or increasing number of health interventions in future years.

Table Q23. Type of output measures produced

Output (sorted from most commonly produced)	Number of countries	
	Platform-based intervention (individual)	Population based interventions (collective)
Total cost by intervention (Unit cost x estimated demand)	5	5
Total cost of packages relative to "do nothing"	5	4
Per capita cost by intervention (Total cost of the intervention divided by total population of the country or other geographical catchment area)	4	4
Average/typical unit costs of an intervention (per user/year or other similar unit)	4	3
Incremental costs (total or per capita cost of delivering the package relative to status quo)	4	3
Cost drivers (%)	4	3
Costs by type of input (for example to fit specific budget configurations or for planning purposes)	4	3
Per capita cost for packages or sub-packages	4	2
Costs by program categories (to fit budget program)	2	2
Indirect health system costs/overheads	2	2
Investment versus operational costs	2	2

Table Q24. Analysis of output measures by category

Number of countries that calculated the output listed	National aggregates	Each intervention	by health delivery platform or health facilities	By disease cat.	By location (geogr.)	By level of care	By type of provider/HR
Unit costs*	2	4	2	2	2	1	2
Average unit costs	3	3	2	2	3	1	2
Total cost	4	3	3	3	1	3	1
Per capita costs*	3	1	2	0	1	0	0
Indirect health-system costs*	2	1	1	0	0	0	0
Cost drivers	4	2	3	1	1	1	1
Specific Input requirements (a)	0	0	0	0	0	0	0
**Total**	**18**	**14**	**13**	**8**	**8**	**6**	**6**
* terminology explained in the survey instructions (page 1 of the survey)							

Table Q25 – Sensitivity analysis

Type of sensitivity analysis done in 3 countries [(a)]	Number of countries
Different scenarios on human resources for health	2
Different scenarios on extent of coverage	2
Sensitivity to different data specification (e.g., using different sources for prices or recommended protocols, using average vs. marginal prices, etc.)	1
Sensitivity to different assumptions (e.g., discount rates, amortization, available technology, linear/non-linear relationships, etc)	1
Different scenarios on investment plans	1
Different geographical expansions	1

(a) Two of the five countries did not perform any kind of sensitivity analysis

Table Q26 – Benchmarking

	DCP3		Literature		Other countries		Other-In country		ALL
**N=Number of countries**	**N[(a)]**	**Score [(b)]**	**N[(a)]**	**Score [(b)]**	**N[(a)]**	**Score [(b)]**	**N[(a)]**	**Score [(b)]**	***Mean score***
Total or per capita costs of packages	2	*6.5*	1	*4.5*	1	*3.5*	2	*6*	***5.1***
Unit costs of individual interventions	1	*4.5*	0	*2*	0	*0.5*	1	*4.5*	***2.9***
Total costs by intervention	1	*3*	0	*0.5*	0	*0.5*	1	*3*	***1.8***
Total costs by platform	1	*3*	0	*0.5*	0	*0.5*	1	*3*	***1.8***
Total costs by disease or program area	1	*3*	0	*0.5*	0	*0.5*	0	*0*	***1***
Cost of population-level interventions	1	*3*	0	*1*	0	*0.5*	0	*1.5*	***1.5***
Costs of intersectoral interventions	0	*0.5*	1	*3*	0	*0.5*	0	*0*	***1***

(a) N includes countries that performed the comparison fully, the score, however takes account of partial treatment and discussions
(b) The index score is calculated by attributing a value of 3 when the comparison was reported as done, 1.5 if it was done partially, and 0.5 if it was discussed but not done. Maximum score is therefore 15 (if all 5 countries performed a given type of analysis)

Table Q27. Principal uses of costing outputs in different types of policy making/decisions

**Scores [(a)]**<br>**Type sorted from most impacted to least**	**Mean**	**Afghanistan**	**Ethiopia**	**Pakistan**	**Somalia**	**Sudan**
Advisory committee meetings	**2.2**	3	3	2	3	0
Prioritisation of interventions	**1.8**	1	3	2	3	0
Implementation scenarios (packages)	**1.8**	1	3	1	3	1
Business case/Resource gaps for funders	**1.8**	2	3	0	3	1
Service delivery planning	**1**	2	0	1	2	0
Health workforce planning	**1**	1	0	0	3	1
Contracting-out (PPPs, NGOs, etc.)	**1**	3	0	0	2	0
Annual budgeting – National/Local	**0.8**	3	0	0	1	0
Medicines, equipment, supplies procurement	**0.8**	1	0	0	3	0
Implementation at different levels	**0.6**	1	0	0	2	0
Infrastructure Investment plans	**0.6**	0	0	0	3	0
Quality evaluation of current services	**0.6**	1	0	0	2	0
Long-term budgeting – National/Local	**0.4**	1	0	0	1	0

(a) The score is obtained by attributing a value of 3 when the exercise had been determinant, 2 when it had been useful, 1 when it was planned to be used, and 0 when it had neither been used nor was planned to be used.

Table Q28. Plans to update the EPHS

**Country**	**Plan to update**	**Modalities**	**Specific constraints**
Afghanistan	Yes	Not determined	Political situation
Ethiopia	No	N/A	--
Pakistan	Yes	Periodic revisions, frequency not confirmed	--
Somalia	Yes	Every 4-5 years or if there a major development in the health sector, particularly resource availability and health system capacity.	--
Sudan	Considered	First need to implement/fund the proposed package	Dynamic context

Table Q29. Guidance on methodology and tools used (sorted from lowest rating)

Rating 0-10<br>adequacy of guidance relative to:	Methodology				Tools				*ALL*
	**Mean**	**Min**	**10s**	**<5**	**Mean**	**Min**	**10s**	**<5**	***Mean rating***
Time required	5.4	0	1	2	5.4	0	1	2	***5.4***
Data required	6.8	0	2	1	7	0	2	1	***6.9***
General usefulness of guidance	7	3	1	1	7.2	5	1	0	***7.1***
Type of output produced relative to anticipated needs	6.8	4	1	1	7.4	4	1	1	***7.1***
General accessibility of guidance	7.8	2	2	1	7	2	1	1	***7.4***
Skills/expertise required	7.8	2	2	1	7.8	2	2	1	***7.8***
Feasibility of different types of analyses	8.4	4	3	1	7.4	0	2	1	***7.9***

Table Q30. Elements to consider when selecting methodology and tools – ratings

From most to least important	Mean rating 0-10
Data issues (requirements, quality, availability, access)	9.6
Flexibility to adapt to country context [(a)]	9
Time required to carry out the exercise	8.8
Skills/expertise required - capacity development needs	8.6
General guidance from international sources	8.2
Prior knowledge of tools used	8.2
Availability/accessibility of specific guidance for the tool	7.8
Number of people needed	7.4
Practical examples of other countries that carried out similar exercises	7.4
Number of interventions to include in costing exercise	7
Financial considerations	5.6

(a) The category was missing in the survey but indicated as "other" and rated high. Highlighted need was the need for flexibility to reproduce results at the provincial/local level

Table Q31. Most appreciated tools and tool features

Country	Tool and specific feature highlighted by the team in cost estimation/analysis and presentation
Afghanistan	Core plus - Excel dynamic sheet – highly recommended by the team. It is developed specifically for costing the PHC packages. Bottom up costing, helps procuring both normative and actual estimates
Ethiopia	**Cost estimation:** OHT is configured in a very systematic way to capture all major aspect of health system cost. Therefore, it helps to produce more realistic estimate. But it is data demanding process.
Pakistan	**Cost estimation:** *The adaptability of excel was very useful*<br>**Analysis and presentation:** *For the HIP tool, the bookshelf and other visual tools were very helpful*
Somalia	**Cost estimation:** OHT Bottleneck and impact analysis<br>**Analysis and presentation:** OHT tools for creating tables and graphs
Sudan	OHT outputs presented in a spreadsheet

Table Q32-33. Output quality and usefulness of costing results in policy making – ratings

N=Number of countries that gave a ranking(a)<br>Mean ratings 0-10	This costing exercise		Potential for future such exercises			
	N	Mean	N	Mean	Number of 10s	Number of <5
General assessment of quality of results	5	7	--	--	--	--
Type of action ranked from most to highest potential						
Contracting-out (PPPs, NGOs, etc.)	4	7.25	4	8.75	1	0
Medicines, equipment, supplies procurement	4	7.5	4	8	1	0
Planning implementation scenarios	5	7.4	5	7.8	2	0
Health workforce planning	5	6.4	5	7.8	2	1
Advisory committee meetings	4	7.5	4	7.75	1	0
Prioritization of health interventions	5	6.8	5	7.4	1	1
Service delivery planning	4	6.25	4	7.25	1	1
Long-term budgeting – national/local	4	5.5	4	6.75	1	1
Implementation at different levels	4	5.5	4	6.5	1	1
Business case/Resource gaps for funders	4	6.25	4	6.25	1	1
Annual budgeting – national/local	4	4.25	4	5.75	1	2
Quality evaluation of current services	4	4.75	4	5.5	1	1
Demand management (through shadow pricing)	1	3	2	5.5	0	1
Infrastructure investment plans	4	4.5	4	5	1	2

(a) The rating was left blank if the respondent had no opinion or did not know. N indicates the number of countries that expressed an opinion of a given type of use

Table Q34. Capacity building by type – ratings

Ratings 0-10<br>Ranked from highest rating for future exercises (potential)	This exercise	Future exercises (mean)		
	Mean rating	Mean rating	10's	>5
On the job training	9.40	9.8	3	0
Additional training requested by individuals in the team based on need	8.33	8.8	2	0
Courses followed abroad	8.33	8.2	2	0
General technical support from development partners	8.00	8.2	1	0
Classes or workshops organised locally	8.00	8.0	1	0
Courses followed locally	8.00	8.0	1	0
Classes or workshops organised abroad	8.00	7.60	2	1

Table Q35. Challenges faced in costing

**Ratings 0-10**<br>**Ranked from most important challenge faced (mean of N countries)**	**N**	**MEAN**	**10s**	**>=8**	**<5**
Time required to carry out the exercise	5	9	2	4	0
Data issues (data requirements, data quality, data availability)	5	9	2	4	0
Lack of information on existing health system capacity/investment needs	5	7.8	2	4	1
Skills/expertise required to carry out the exercise	5	7.4	1	3	0
Treatment of investment costs	4	7.25	1	2	0
Number of people needed to carry out the exercise	5	7	0	2	0
Availability or timeliness of capacity training	5	7	1	2	0
Lack of practical examples from other countries	5	6.6	0	2	1
Availability/accessibility of specific guidance for costing tools	5	6.4	1	1	0
Difficulty in explaining results to decision makers	5	5.6	0	0	0
Covid-19 related issues	5	5.6	1	2	1
Coordination/timing issues, including with other teams	5	5.2	0	1	2
Understanding of terminology	5	4.8	0	1	2
Other added: Specific country context (political situation)	1	4	0	0	1

Table Q36-38. Open suggestions of country teams

Suggestions for improvement and open comments (all feedback is included)
**Capacity building-related comments:**<br>- *Conducting a full training programme on the costing tool for the research team.*<br>- *Training on the use of OHT and interpretation of the results should be part of initial training of the MoH.*<br>- *Any costing tools should be accessible and usable by public health specialists and other ordinary health professionals with targeted and minimum training. This does not mean the country should not have specialized health economists and other experts needed for the design, selection of interventions and costing and cost effectiveness analysis*<br>- *Train more health and ministry of finance professionals to better understand the cost of health care and its contribution to the economic growth through increased productivity, absenteeism of schools and workplaces etc*.<br><br>**Knowledge sharing and stakeholder involvement**<br>- *Now that intervention sheets have been developed, -they could be reused for future exercises and shared with other teams, particularly relating to input requirements.*<br>- *Ensure all key stakeholders are engaged from the design to the final stage*<br><br>**Methodology and costing Outputs**<br>- *Calculate cost estimate by level of delivery*<br>- *Calculate different costs for different care settings*<br>- *Costing was undertaken by intervention rather than at programme level and by care setting. This is too reductionist for the purpose of priority setting and assessing funding requirements and funding envelopes*<br>- *Primary health care would adopt more of a capacity driven approach based on units of capacity (such as a clinic, polyclinic etc).*<br>- *Whatever exercise is used, the results need to be consolidated by care programme and care pathway to ensure they make sense and provide a coherent range of interventions for any given presenting condition or health risk.*<br>- *Produce the costs of surgical interventions in more details*<br><br>**Validation**<br>- *If more time could be allowed, the quantities should be reviewed in greater detail. Ideally, some more validation of both the costing and the intervention description sheets could be useful.*<br><br>**Other open suggestions**<br>- *Countries could prepare a road map describing the costing approach before starting the main costing exercise. The costing plan can be a short description of the team composition, what methods to use, and data sources.*
