# Supplementary material for: Using costing to facilitate policy making towards Universal Health Coverage: findings and recommendations from country-level experiences
Source: BMJ Glob Health. 2023 Jan 18;8(Suppl 1):e010735. doi: 10.1136/bmjgh-2022-010735 (PMC9853124; doi:10.1136/bmjgh-2022-010735)
Supplement: Supplementary data [file bmjgh-2022-010735supp003.pdf]

SUPPLEMENT - ANNEX 3 (SA3): BASIC TERMINOLOGY IN CONTEXT

UNIT COSTS: conceptually very different from a per capita cost (see below), they measure the cost of providing a specific health service (intervention) to one user. The term is meaningful for healthcare interventions on the person (as opposed to population interventions). The unit could differ (per visit, per hour, per bed/day, etc.). The unit cost may be calculated differently depending on intended use. Including a fair share of fixed and overhead costs is important when the unit cost is used to determine user fees or shadow prices (i.e. what it cost to society to provide a service even the service is delivered for free to users). It may be calculated without overhead costs in specific cases, such as when it is used to determine provider payment in cases when the provider is not responsible for overheads. Unit costs can also be used as a component to estimate total and per capita costs of specific packages of services. In such cases, it will need to be weighted using estimates of expected demand for the service (or target populations). When looking at the costs of population interventions, the term “unit costs” must represent how much it costs to provide the intervention itself since it cannot be reduced by reducing the number of beneficiaries. It is possible that population interventions reach some populations and not others; for example, when only people from one region can benefit while others are excluded. In that case, the unit cost is the cost of providing the intervention for a specific population. Even if the term “unit cost” may be used for population interventions, it is not appropriate to compare unit costs of population-based interventions to unit costs of healthcare interventions delivered on an individual basis.

TOTAL COSTS: they are the result of multiplying unit costs and quantities demanded (or delivered). For the purpose of costing health packages, this is done for each intervention (total intervention cost) and then aggregated across interventions to get the total cost of a package. To calculate the total cost of delivering a given healthcare intervention, one needs to take account of population in need as well as level of coverage over a given unit of time (generally one year if not otherwise specified). It is important to match the units before aggregating across interventions. One also needs to pay attention whether fixed and overhead costs are included in intervention costs or not. If fixed costs and overheads are not included in individual intervention costs and if delivering the package entails program costs in addition to what is borne by the health system, these costs need to be added. As for

population-based interventions, their annual cost may be added to the total to obtain the cost of packages that include both personal healthcare and population-based interventions.

We note that when looking at the costs of population-based interventions, the total cost of the intervention is also equal to its unit costs, as defined above. The cost per person affected by the intervention is only meaningful to compare among population-based interventions if the affected populations are very different (for example when one would compare an intervention related to HIV to one related to preventing type II diabetes or tobacco smoking).

PER CAPITA COSTS: they are calculated as total costs divided by total population (country or specific area of reference/catchment area). Per capita costs are generally reported instead of total costs for ease of interpretation and comparison across countries or other catchment areas. Per capita costs should not be confused with unit costs, particularly for individual healthcare interventions when it is very rarely the case that the entire population uses the same service.

INCREMENTAL COSTS: they are calculated relative to a baseline of what is already being spent. In the exercise of costing UHC packages of health services, incremental costs are useful to understand what additional financing is needed to provide more services or expand coverage. This is particularly useful for budgeting purposes. However, it is important to exercise caution when comparing incremental costs across countries given that they all start from a different base.

INDIRECT (HEALTH SYSTEM) COSTS: In health care, the term is used to designate all costs supported by the health system in relation to providing healthcare services in general but not directly related to patient care or specific interventions. These costs are sometimes called ***"common costs",*** as they are the same across different types of services (although they may differ across different platforms). Examples of indirect costs are general administration and overhead costs, information technology, some human resources costs (if not included as ingredients in service provision description sheet), monitoring and evaluations, etc. For the purpose of carrying out costing exercises, what matters is that these indirect costs do not vary directly with the level of provision of individual services. What is included in indirect costs and how these costs are incorporated in the estimation could become an important issue in costing methodology.
